# Land-Use and Socioeconomic Change, Medicinal Plant Selection and Biodiversity Resilience in Far Western Nepal

**DOI:** 10.1371/journal.pone.0167812

**Published:** 2016-12-09

**Authors:** Ripu M. Kunwar, Kedar Baral, Prashant Paudel, Ram P. Acharya, Khum B. Thapa-Magar, Mary Cameron, Rainer W. Bussmann

**Affiliations:** 1 Department of Geosciences, Florida Atlantic University, Boca Raton, Florida, United States of America; 2 District Forest Office, Baitadi, Nepal; 3 Tropical and International Forestry Program, Georg-August University, Gottingen, Germany; 4 Practical Solutions, Kathmandu, Nepal; 5 Department of Ecology and Evolution, Stony Brook University, New York, New York, United States of America; 6 Department of Anthropology, Florida Atlantic University, Boca Raton, Florida, United States of America; 7 William L. Brown Center, Missouri Botanical Garden, St. Louis, Missouri, United States of America; Institute of medical research and medicinal plant studies, CAMEROON

## Abstract

Indigenous plant use-systems have evolved under, and constantly adapted to human and non-human impacts. In the last decades however, increasing socioeconomic and cultural transformations, including land-use change, outmigration, globalized markets, the introduction of new species, and climate change have led to a decreasing availability of indigenous resources, and are ultimately leading to a reduction of local use-knowledge. Participant observations, discussions, *walks-in-the-woods*, semi-structured interviews and informal meetings were carried out in 12 villages of far western Nepal between 2011 and 2015 to assess how sociocultural changes have affected the sustenance of indigenous systems and local biodiversity, when compared to studies carried out in the previous decades. Our findings show that there were no statistically significant differences in subject variable means, but differences were relatively important to plant parts-use and plant growth-forms (*p* = 0.183 and 0.088 respectively). *Cissampelos pareira*, *Acorus calamus*, *Calotropis gigantea* were found to have the greatest relative importance, whereas *Ageratina adenophora*, *Melia azedarach*, *Carum carvi* were most important based on use values. Among them, *C*. *pareira* and *A*. *adenophora* were introduced. The spatial distribution of species collected for medicine showed that all habitats were important for collection however, habitats close to villages were more favored. The use of non-indigenous and easily available species and more accessible habitats is becoming more prevalent as primary forests become increasingly overexploited, indigenous species become limited, and sociocultural cause of land use change expand. The utilization of indigenous and non-indigenous species and nearby habitats, although possibly affecting the quality of medicinal species, nonetheless reveals the dynamism of indigenous medicines as an adaptive asset mitigating human and non-human environmental changes.

## Introduction

Indigenous medicine represents a socioculturally coherent [1], resilient and valuable resource [2] that is sustained locally through the use of indigenous medicinal plants and their associated knowledge and considered as an active constituent of global medical plurality. Medicinal plants are an integral part of indigenous medical systems and livelihoods, and have long been collected, consumed, and conserved by indigenous populations, leading to a wealth of accumulated indigenous knowledge [3,4,5]. Indigenous knowledge is often hailed for its versatility to recognize and respond to livelihood changes [6], yet such knowledge can be transformed by ecological and sociocultural evolution [7]. Some indigenous medicinal plants are severely threatened, and various alterations in species composition and distribution are taken place due to changing climatic gradients [8, 9, 10], land-use patterns [11,12,13] and socioeconomic and cultural changes [1,14]. Indigenous people’s land use practices in general and medicinal plants in particular are both susceptible to environmental changes [15], with each producing alterations in the other [11].

Reductions in medicinal plants and associated indigenous knowledge can compel local people to integrate more non-indigenous resources in their pharmacopoeia [16,17,18], and to access second-growth habitats [19] in their indigenous use-systems. As old-growth forests become overexploited [20,21], readily accessible and pharmacologically rich secondary forests [22,23] are gaining importance for medicinal plant collection. Indigenous use-systems have now evolved after the shock of original contact [18,24], with decreasing availability of indigenous resources, and the introduction of new species [25,26], socioeconomic transformation [7], indigenous knowledge effacement [26], and climate change [27]. Phillips and Gentry [28] and Thomas *et al*. [29] proposed ecological apparency hypothesis that the most common and accessible species and habitats are those which are used and about which ethnobotanical knowledge is created. In the light of rapid changes in accessibility in species and habitats we hypothesize that, independent of whether a plant or habitat is indigenous to an area or newly introduced, commonness and accessibility will drive use and valuation, aided by sociocultural change. In order to test this hypothesis we analyzed the collected species and sites and their importance to ethnobotany and indigenous medicine in far western Nepal, and assessed local peoples’ attitude towards the conservation of these resources at the nexus of climate change and sociocultural transformation.

## Materials and Methods

### Study area description

**Fig 1:**
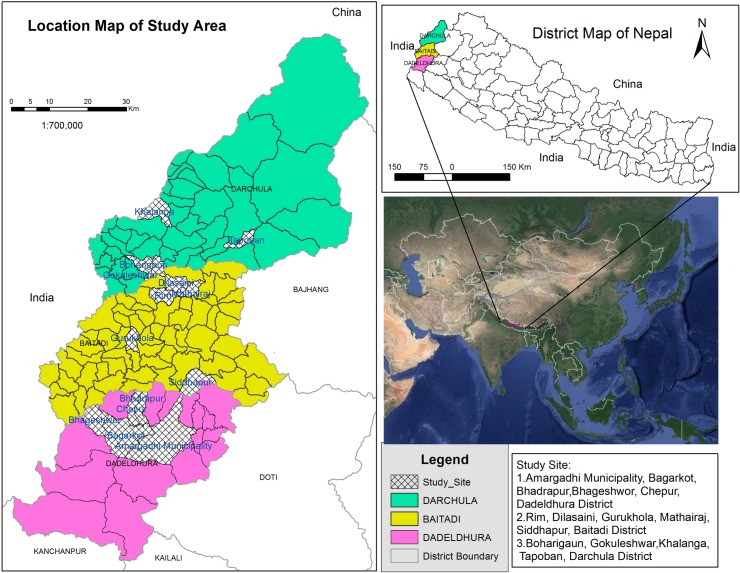
Study area.

The study area represents the westernmost area of Nepal (Fig 1), including Baitadi, Dadeldhura and Darchula district (29° 01' N to 30° 15' N / 80° 15' E to 81° 09' E). A varied topography [30,31] with elevations ranging from 257 to 7,132 m harbor diverse forest types, from tropical humid forest to alpine *Betula*-*Rhododendron* scrub [32]. The climate ranges from tropical in the mid-elevational hill districts Dadeldhura and Baitadi to alpine in the higher reaches of mountainous Darchula district [33,34], with an average annual rainfall of 2,326 mm (R^2^ 0.08) and an average temperature 18.15°C (R^2^ 0.63). Darchula has about 25% forest cover, whereas Baitadi has 40% and Dadeldhura has 60%. The study districts contain six types of forest ownership and management namely national, protected, community, leasehold, religious, and private. The largest community managed forest cover is found in Baitadi (69% of total forest cover), followed by Darchula (34%) and Dadeldhura (20%).

Baitadi and Darchula districts are part of the Kailash Sacred Landscape (KSL), a collaborative trans-boundary landscape program initiated in 2010 among China, India and Nepal to conserve ecosystems and biodiversity, and to promote sustainable resource management [35,36]. Numerous sacred religious sites located near high-altitude lakes and snow-covered peaks across the three countries characterize KSL. The holy Mt. Kailash and the adjacent Lake Mansarovar are the most important of these, and have been pilgrimage destinations for followers of Hinduism, Buddhism, Jainism, Sikhism, and Bon for several millennia [37]. Dadeldhura district serves as the southern buffer for KSL. Much of the KSL consists of steep and rugged terrain, and only 7–21% of the land is suitable for farming [38], resulting in regular food deficiency [39]. Upper Darchula district is originally known for growing amaranth [40] and is a part of the relict hemp cultures [41]. More than two thirds of the population still relies on indigenous livelihood strategies such as animal husbandry, transhumance, seasonal crop production and collection, and the use and trade of medicinal plants [42,43,44,45]. The limited livelihood options, together with modern changes in lifestyle as a result of globalization, and droughts and erratic weather patterns from climate change, all threaten the sustainability of these mountain communities, the landscape, and its biodiversity [37].

The major caste groups of the study area are Kshetri (>50%), Brahman (20%), Thakuri (7%), Kami (10%) and Sarki (8%). Kumahi Brahmans were considered the first Aryans reaching Nepal in 1100 AD, and settled in the western parts of the country; however the pre Vedic and Vedic Aryans were considered the early settlers [46]. The pre Vedic and Vedic Aryans (Urus) might have entered Tibet and far western Nepal via the Urai pass at Bajhang district in far western Nepal. The trek route to Kailash Mansarovar via Urai pass and Lipu lek (Darchula) is still popular today [47]. Therefore, the advances from Aryans into the western parts of the country are considered early accounts of human civilization, and forest and plant exploitation in Nepal. These ‘heritage routes’ and the remnants of their once-flourishing trade add to the beauty and rich cultural history of the region [37]. The fossilized tooth of a *Ramapithecus* found in 1980 in the Tinau (Butwal) area of west Nepal dated to be the second oldest in the world at 9.0–9.5 million years [48], substantiates the prehistoric use of the area as a prime habitat of *Ramapithecus* [49]. A few remote villages of Dadeldhura district are occupied by the forest-dwelling *Raute* tribe, members of which live in and travel between the Siwalik hills in the south and mountainous highlands in the north, maintaining their livelihood by nomadic hunting and plant foraging [50,51].

### Data collection

This study is a part of larger effort to document the ethnobotany of the Himalayan and Kailash Sacred Landscape conducted since 2004. Both oral and written consents were obtained for study. Oral consents were obtained from participating communities with the help of local assistants. Written consents were granted from district forest offices. After establishing prior informed consent with the participating entities, participant observations, discussions, *walks-in-the-woods*, semi-structured interviews and informal meetings were conducted. Four field visits of about 20 days each were conducted between 2011 and 2015. An IRB approval was obtained from Florida Atlantic University to carry out field study in 2015. A total of nine participatory *walks-in-the-woods* were made along forest trails around 12 villages (Dilasaini, Guru khola, Maithraj, Rim and Siddhapur (Baitadi), Bagarkot, Bhadrapur, Chipur and Amargadhi (Dadeldhura), and Bohori gaun, Gokule, and Tapoban (Darchula) (Fig 1). These villages were randomly selected from 106 in the three districts. Elevation of the trails ranged from 695 m to 2,321 m in Baitadi, 750 m to 2,564 m in Dadeldhura, and 644 m to 3,004 m in Darchula. In each *walk-in-the-woods*, all useful species, with special focus on medicinal plants, were listed, vouchered and discussed with the participants [52] following both inventory [53] and visual cues [54].

Nine discussion meetings were held during the evenings while staying with local communities, and sometimes with tea vendors in the mornings with the help of local assistants, using an open Nepali language questionnaire (S1 File). Four informal meetings were conducted during the field visits to collect information about newly introduced species at particular sites. Conversations with healers and residents were based on the common objective–as suggested in the International Society of Ethnobiology Code of Ethics [55]–to increase knowledge regarding natural remedies and develop educational materials of local interest. There were altogether 54 discussants (50 male, four female), 52 from upper caste groups and only two from lower caste under-privileged groups [56,57,58,59] which represented traditional healers, community forest users, and elderly persons of the villages, aged 41 to 85. All plant species were first field-identified using vernacular ethnotaxonomic information, verified using literature [60,61], and validated and housed at the National Herbarium and Plant Laboratories (KATH), Lalitpur, Nepal.

### Data analysis

Previous studies in this area [5,62,63] were used as a database to retrieve the biogeographical distribution of species in the region. Species origin, usefulness, use value index, growth form, parts used, etc. were taken as sub-variables for analysis. We considered species as indigenous when they grew naturally in the area or had long been cultivated. Globally, native and non-native status are generally determined by one (or both) of two concepts: (1) presence in an area before an arbitrary cut-off date imparts native status and (2) human-mediated movement of individuals results in non-native status [64]. Matching information from at least three respondents was counted as a common response for quantitative analysis [65]. The use-value (UV) index [28], adapted by Albuquerque *et al*.[66], was used to calculate citation of plants during interviews. It is calculated as UVc = ∑U / ns, where U is the sum of the total number of use citations by all informants for a given species, divided by the total number of informants (ns). The UV, which is based on the number of uses and the number of people that cite a given plant, is considered important within the ethnobotanical community for helping describe the relative ‘saturation’ of a plant’s life in the human community [67]. The fidelity level (FL) value was calculated to estimate the healing potential of each medicinal plant mentioned. The FL is defined as the ratio of the total number of informants that independently cited a specific plant use (Np) and the total number of informants (N) that cited the plant for any use. It was calculated as FL = (Np / N) x 100.

Each ailment was classified according to its level of species redundancy: highly redundant (≥15% species of total species employed for a particular ailment category), redundant (15% < number of species ≥ 5%), and not very redundant (number of species < 5%) following [68]. The relative importance index (RI), indicative of the level of diversity of medical applications, was computed for each reported medicinal plant by using the formula RI = NP + NCS [17], where NP is obtained by dividing the number of properties (reported to be useful for specific ailments) attributed to a species divided by the total number of properties attributed to the most versatile species (the species with the highest number of properties). NCS is the number of body systems (ailment categories) treated by a given species divided by the total number of body systems treated by the most versatile species. Species with RI value 2.0 (the highest possible value) are those with the highest diversity of medicinal use. The RI value is derived from the number of health indications (suggestive of pharmacological properties) for that species and from the number of ailments that it is used to treat. We performed a simple evaluation of UV and RI in order to assess the correspondence between them. For better interpretation, the mean values of RI and UV were compared among the variables: plant growth form (herb, shrub, tree and climber); plant origin (indigenous, non-indigenous, total); parts used (roots & rhizomes, flowers & fruits, leaves, woods & seeds, and others); district (Baitadi, Dadeldhura and Darchula); and land-use type (undisturbed forest, disturbed forest, transition land, and agriculture land) using a one way analysis of variance (ANOVA). Simple linear regression was used to show the relationship of age and knowledge of useful medicinal plants. Prior to ANOVA, the data were tested for homogeneity of variance. Probability values of *p* <0.05 were considered to be statistically significant [69].

## Results

### Medicinal plants

The inventory of usefulness of species in the 12 villages showed that there were 74 important plant species and products used as ethnomedicine with more than three mentions (S1 Table). Among the districts, the participants from Dadeldhura district used the least number of species (50), including the least number of indigenous ones (35), whereas the highest number of species (66, 50 indigenous and 16 non-indigenous) were found to be used in Baitadi. Statistically, there were no significant differences in the means of UV and RI of medicinal plants among the three districts, four land-use types, and medicinal plant origin, but the relative differences with regard to plant parts used and growth-forms at ANOVA (*p* = 0.18 and 0.08, respectively) were important (Table 1).

**Table 1:** Descriptive statistics and ANOVA based on UV and RI of species.

Variables	Categories (N = 74)	Use Value (UV)				Relative Importance (RI)			
		Mean, St. dev	St. error	f value	*p* value	Mean, St. dev	St. error	f value	*p* value
District	Dadeldhura (n = 50)	0.155±0.253	0.035	0.08	0.91	0.211±0.085	0.012	0.03	0.96
	Baitadi (n = 66)	0.143±0.227	0.028			0.208±0.082	0.010		
	Darchula (n = 56)	0.136±0.237	0.031			0.211±0.085	0.011		
Land-use	Primary forest (n = 23)	0.130±0.180	0.037	0.18	0.90	0.181±0.075	0.015	0.47	0.70
	Secondary forest (n = 35)	0.114±0.161	0.027			0.202±0.070	0.110		
	Transition land (n = 40)	0.133±0.242	0.038			0.204±0.083	0.130		
	Farmland (n = 15)	0.161±0.263	0.068			0.193±0.090	0.023		
Species origin	Indigenous (n = 57)	0.127±0.182	0.024	3.97	0.29	0.212±0.087	0.021	3.95	0.55
	Non-indigenous (n = 17)	0.194±0.350	0.085			0.198±0.080	0.010		
Plant growth-forms	Herbs (n = 43)	0.108±0.246	0.037	0.27	0.84	0.194±0.081	0.012	2.26	0.08
	Shrubs (n = 10)	0.148±0.202	0.064			0.195±0.091	0.028		
	Trees (n = 15)	0.144±0.227	0.058			0.196±0.065	0.016		
	Climbers (n = 6)	0.209±0.198	0.081			0.283±0.080	0.032		
Plant parts used	Roots & rhizomes (n = 27)	0.990±0.112	0.021	1.66	0.18	0.202±0.091	0.017	0.16	0.91
	Flowers, fruits, and seeds (n = 20)	0.100±0.172	0.038			0.193±0.075	0.016		
	Leaves, stems, barks and woods (n = 23)	0.229±0.344	0.071			0.205±0.078	0.016		
	Others-mixed (n = 4)	0.152±0.207	0.103			0.222±0.091	0.045		

Among the important 74 ethnomedicinal plant species, 43 were herbs, 15 trees, 10 shrubs and 6 climbers. The frequent use of herbaceous species could be a result of their relative abundance as compared to trees and shrubs with their higher percentage of alkaloids, phenolics and cyanogenic glycosides [70]. Frequent use of herbs in ethnomedicine supports a chemical view of the ecological apparency hypothesis, where more hidden plants (herbs) reveal a qualitative strategy investing in compounds with medicinal and low molecular weight [71]. Other plant forms like climbers and shrubs were also therapeutically competent (UV of climbers 0.209±0.19; shrubs 0.148±0.20; trees 0.144±0.22; herbs 0.108±0.20). A total of 35 species exhibited both ethnobotanical and ethnomedicinal values to humans. We found a total of 72 genera and 42 families, with Asteraceae, Fabaceae and Apiaceae being the dominant families, contributing 8, 4 and 4 species, respectively. The prevalence of Asteraceae and Fabaceae in local pharmacopoeias was also reported in other studies [5,17,72,73]. We found 17 (23%) non-indigenous medicinal plant species representing 11 families, which is within the range (12–44%) elsewhere reported [74,75,76]. Seven out of 17 were from Asteraceae family and 12 were collected from lands with ruderal characteristics.

The total number of uses (mentions) for a species for a particular ailment (FL) was found to be similar for non-indigenous species *Ageratina adenophora* (FL = 100% for bleeding control) and indigenous species *Melia azedarach* (FL = 100% for fever). Other species with relatively similar responses of more than 75% FL were *Aloe vera* (non-indigenous, 79%) for burns, *Carum carvi* (indigenous, 83%) for cough and cold, *Angelica glauca* (indigenous, 88%) for indigestion. Other indigenous species with higher FL values were *Cuscuta reflexa*, *Euphorbia royleana*, *Paris polyphylla*, and *Bergenia ciliata* with FL >20%. Higher FL and RI values indicate species regarded as having good healing potential.

A total of 51 ailments under 13 body systems [77], were treated by 74 plants and products, with diarrhea and dysentery, fever, and stomachache treated the most redundantly (with 14, 13, and 11 different species used for treatment, respectively). It was found that 36 ailments were “not very redundant”, and 12 were “redundant”. Cough and cold, skin diseases, and tonics were treated by nine species. Eleven ailments were treated by any one of five local species, and 20 ailments were treated with only one species. A total of 36 locally available medicinal plants were useful in treating digestive system disorders.

### Use values, relative importance and collection

**Fig 2:**
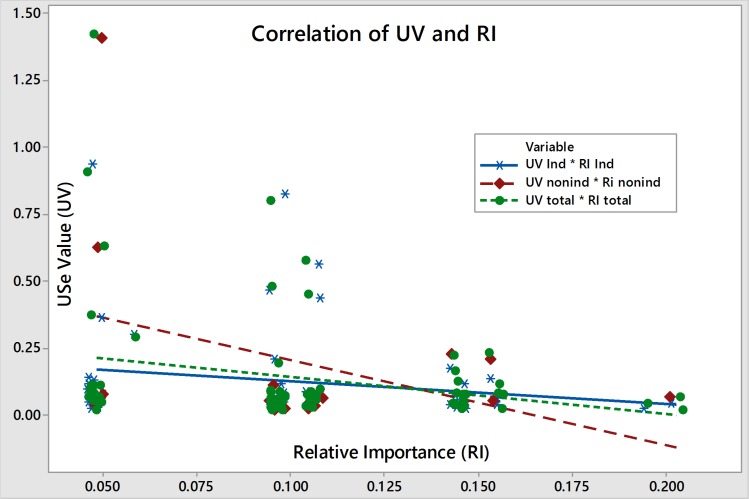
Correlation of RI and UV by species origin.

Plants found to be medicinally important as measured by RI included *Cissampelos pareira*, *Paris polyphylla*, *Acorus calamus*, *Adiantum capillus-veneris* and *Cuscuta reflexa*. Those found important based on UV included *Ageratina adenophora*, *Melia azedarach*, *Carum carvi*, *Aloe vera* and *Angelica glauca* (Table 1). Among the species, *Cuscuta reflexa*, *Paris polyphylla*, *Melia azedarach*, *Carum carvi*, and *Acorus calamus* were indigenous while *Adiantum capillus-veneris*, *Ageratina adenophora*, *Aloe vera*, *Cissampelos pareira* were non-indigenous. We found that RI and UV and their relations were specific to species origin. The scatterplot shows that RI and UV were homogeneously distributed and negatively correlated (- 0.24, *p* = 0.036) for indigenous species, whereas the values for non-indigenous species were highly ranged, heterogeneously distributed and negatively correlated (Fig 2). We concluded that RI and UV are independent, specific to species origin, and based on their usefulness to treat infirmities and consensus of participants.

**Fig 3:**
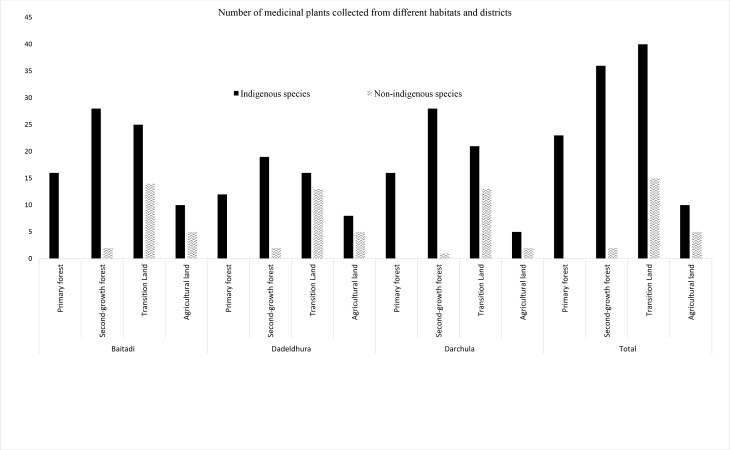
Number of medicinal plant species collected from different habitats and districts.

Our results show that non-indigenous species are abundant in areas close to human dwellings (15 out of 17 are found only in transition lands and agricultural lands) and their richness decreased along the distance gradient from home, with only two in secondary forest and none in primary forest. The primary forests were second to transitional land and second-growth forests as collection localities for indigenous medicinal plants in far western Nepal (Fig 3), and were especially visited by healers. Twenty-three indigenous medicinal plant species were recorded from primary forests, while transition land harbored 40 medicinal plant species, including 15 non-indigenous. The distribution of collected species showed that all habitats were important for collection, but accessible habitats were more favored. The mean values of both UV and RI were higher (0.133±0.24, 0.204±0.08) for transition land than primary forest (0.130±0.18, 0.181±0.07). Old-growth primary forest remnants, located farther from homes, roads and agricultural lands, were less accessed. These findings suggest that traditional medicinal plant selection and collection from primary forests, themselves reduced in size over time, are gradually replaced by relatively easier and physically less demanding ways of selection and collection. Furthermore, lay-persons held less knowledge (5.63±1.19) of medicinal plants than healers (6.2±5.17), *p* = 0.32. However, we did not find a significant knowledge-age relationship.

## Discussion

### Medicinal plants, knowledge and culture

Collection and use of non-indigenous medicinal plants for indigenous medicines is not a new phenomenon [78], nor unique to rural areas of far western Nepal [79,80]. While previously non-indigenous species were often overlooked in favor of indigenous ones or not recognized as important [81], they are now gaining importance in local and indigenous medicines [17,82,83] because of their availability and often easy accessibility and preferences. Some other factors driving the introduction of non-indigenous species are population growth and land-use change. The annual population growth in the districts (about 2%) was outpaced by the land-use change (forest cover loss 2.36%). Species introduction for specific purposes first by indigenous and later by diaspora communities could be a potent cause of transformation of indigenous knowledge systems. The number of people residing in rural areas decreased from 95% in 1951 to 90% in 1981 [84], and 80% in 2011 [85]. As a part of a national trend, a continuous outmigration for menial work in India [86,87], and a greater percentage of absentee population of 7.51%, higher than the national average of 7.23%, clearly foment a decline in the number of healers and indigenous knowledge. Decreasing knowledge of indigenous species aids incentives to non-indigenous resources introduction [88]. The effects of outmigration are multi-fold: agricultural fields are left fallow due to lack of labor [89] resulting in decreased productivity [90], and non-indigenous species are spreading. Due to changes in human population structure and sociocultural transformation, a considerable amount of indigenous knowledge of plant resource use is at risk of being lost. We found that the indigenous medicine of far western Nepal is a mosaic constituted by both indigenous and non-indigenous resources. Although the indigenous medicinal plants were the original basis of traditional medicine, non-indigenous species by now compose 23% of the medical repertoire, and further introductions are increasingly likely.

Medicinal plants with ruderal life history characteristics were frequently foraged in the study area and tend to be more tolerant of habitat disturbance, degradation, and cattle browsing [12,91]. Accounts of introduction and uses of non-indigenous invasive species (*Ageratina adenophora*, *Ageratum conyzoides*) date back decades [79,92,93]. The decade-long commercial collection of medicinal plants in Baitadi and Dadeldhura districts shows an increase in harvesting non-indigenous species [92] and similar observations were made in southwest China [94]. The introduction of non-indigenous species was accelerated in our study area when national (primary) forests of the districts were overexploited and community forests were promoted in 1992, although community forest management was instituted in the districts since 1979 [95]. Greater collection from private and accessible forests in Dadeldhura district has been previously reported [86].

The use of non-indigenous species and human-derived landscapes for livelihood is gaining ground as old-growth primary forests become overexploited [20,21], indigenous species decline, traditional practices are discarded and knowledge declined. A ten-year overview on trade data of medicinal plants from the studied districts showed that there were 23 species in trade in 2003 and only 14 in 2013, while the volume of trade had slightly declined [96,97]. Twelve species and products mostly found in remote and old-growth forests such as *Ephedra gerardiana*, *Paris polyphylla*, *Berberis asiatica*, *Dioscorea deltoidea*, *Selinum wallichianum*, *Neopicrorhiza schrophulariiflora*, *Pistacia chinensis*, *Alstonia scholaris*, *Taxus wallichiana*, *Curcuma zeodaria*, *Aconitum ferox* and various lichens had ceased being traded by 2013, while *Eulophia dabia*, *Persea odoratissima*, and *Moringa oleifera*, all subtropical and common in transition areas and disturbed forests were introduced. This indicates that indigenous forest species are gradually replaced by species growing in ruderal areas, consistent with the findings of Bista and Webb [92] and Xu and Wilkes [94]. The species no longer in recent trade were probably due to their overexploitation, people’s reluctance to go to remote and distant areas for collection and or their market price decline. Linear regression analysis showed that traditional healers recounted more useful medicinal plants (6.2±5.17) than elders (5.47±0.26) and other lay-persons (5.63±1.19), but that knowledge of the number of useful medicinal plants was homogenous and insignificantly linked to age, consistent with the findings of Ladio and Lozada [98,99], and contrasting to other findings [100,101]. This could partially be attributed to elderly people’s unwillingness to fully and completely share what they regard as secret information, a common practice [12,102].

Traditional healers are considered experienced and knowledgeable in identifying and collecting more useful plants than other groups of a society [103]. They believe that indigenous medicinal plants and high altitude, pristine and old-growth forests produce quality medicines, and these dedicated healers reported traveling sometimes long distances in search of what they believed to be high quality resources. The distant and undisturbed sites are often cited by traditional healers as refuges for quality products supported by studies from adjoining areas of India [104,105] and other parts of the world [106,107] where a higher number of indigenous species with medicinal usage are being used at remote and higher altitudes. People in higher elevation villages know and use more medicinal plants than people in lower villages [102,108]. However, Toledo *et al*. [109] found that distant and primary forests were tended to be concentrated for food and timber and the secondary forests for medicinal and other non-timber products. Although indigenous plants are important and used for indigenous medicines, the use of non-indigenous species and habitats were more preferred and may soon overtake the traditional base at the expense of loss of the indigenous forests and species and with the trade-offs of sociocultural changes. The richness of useful plants at low elevation and accessible sites could be possibly due to favorable environmental factors or greater access of human populations, resulting in higher pressure on use of any plants in lower elevations. Therefore, our explanation needs further discussion because the themes of priority on indigenous or non-indigenous resources are based on scale and context.

Digestive system disorders were more frequent and their treatment was possible with 36 locally available medicinal plants, consistent with earlier studies [5,110]. The high use of medicinal plants to cure digestive system disorders could be attributed to the high preponderance of that ailment in the area. Diarrhea and dysentery, the most common illness reported in far western Nepal [85], are often caused by people drinking contaminated water, eating improperly stored spoiled foods, and conditions of poor general nutrition. Health is further jeopardized by food deficiencies [34,39]. The health and development index, partly a measure of nutritional status, ranks the study districts (Baitadi, Darchula and Dadeldhura) 66, 62 and 58 among 75 districts of the country [111]. For people suffering from diarrhea and dysentery, indigenous medicines were the first choice.

### Plant growth-forms and collection sites

Plant growth-forms significantly relate to the biogeographical origin and the herbs are found more exotic in origin [106]. Consistent with these findings, we found 58% of all medicinal plants used were herbs, with 80% being non-indigenous species. One very medicinally important group of herbs is the Asteraceae family. Asteraceae in temperate regions are highly herbaceous [112]. This family, which includes eight species, seven of which are introduced and herbs, is widely used because of its strong organoleptic properties [113,114] and higher tendency to grow in secondary habitats near residential areas [115,116]. The widespread collection and use of the species of family illustrates the direct relationship between availability and its cultural importance because people get more opportunities to see, learn about and evaluate the species growing closest to their home [117]. Weeds are also amply represented in folk medicines because they are growing abundantly at ruderal and secondary habitats, are easy to harvest, and are frequently rich in bioactive compounds [22,91,118]. Quantitative analyses (UV and RI) showed that both the indigenous species (*Cuscuta reflexa*, *Paris polyphylla* and *Melia azedarach*) and non-indigenous species (*Ageratina adenophora*, *Aloe vera*, and *Cissampelos pareira*) are important in indigenous medicine in far western Nepal and are candidates for further phytochemical assessment. Etuk and Mohammad [119] stated that plants with higher UV and RI are more likely to be biologically active. UV has also been associated with conservation because the highest UV species receive the greatest harvesting pressure [67,120]. We can deduce that the indigenous species with higher UV (*Cuscuta reflexa*, *Paris polyphylla* and *Melia azedarach)* show potential for ethnobotany and conservation.

We consistently observed that people preferred to collect plants that were easily harvested nearby. It was found that such plant collecting gave less consideration to quality [121] and more importance to subsistence, time, accessibility and familiarity. High use value of *A*. *adenophora* (1.42) and *M*. *azedarach* (0.92) from highly accessible area is related to the fact that those species are highly known and frequently collected, consistent to the findings of Thomas *et al*.[117]. Higher UV, RI and species richness of medicinal plants at human-derived landscapes revealed that the species richness and use values were positively correlated, as found in China [122,123] and the accessibility and familiarity were more concerned. Accessibility was a primary consideration for generalists because they often initially collect plants from easier and common sites and resort to less accessible and specific areas if their first attempts do not meet the expectations. The species collected from a site thus could be attributed to ease of collection in terms of plant growth-form, accessibility, familiarity, availability and time. Therefore, we should say the current folk therapy traditions in our study area are not traditional in the fullest sense. Traditional tends to imply a static remnant of the past [124]. We found that local people used the plants on the basis of availability and accessibility in the area and not always according to their cultural values. However, the cultural values were more decisive in plant use and collection in high altitude areas of China [125].

### Theoretical relevance

Frequent foraging of secondary habitats like transition areas, roadsides and fringes of agricultural lands characterized with anthropogenic interferences, moderate disturbances and prolific growth of non-indigenous plants [71] substantiates the application of ecological apparency hypothesis. The most common and accessible species and habitats are more valued in ecological apparency hypothesis [28,29]. The preference for nearby habitats and species is rooted in optimum foraging theory [126]. The theory presents cost-benefit trade-offs in foragers’ choice, applying and analyzing in ethnobotanical research the required costs (time to access the distance and time to manage the produce) [127], and procured benefits [128]. MacArthur and Pianka [129] expected that the most productive areas with higher availability of plant resources (areas with higher energy) will have a larger amount of extraction events and will be more recognized locally, receiving, therefore, an increased amount of use mentions. Therefore, the distance and time to be traveled to obtain a resource determines the choice of the site to be visited, and the energy (management) to be invested to obtain a product determines the species selection. It is more likely that closer resource areas receive greater attention in plant resource collection (less time required) and are subjected to a greater number of extractions as substantiated by the ecological apparency hypothesis. The hypothesis is further strengthened when there are a number of species with redundant utilities. Diarrhea and dysentery and fever were treated by 14 and 13 species respectively which was in line with the preponderance of these ailments in the area [85]. Higher numbers of redundant utility species will correlate with higher degrees of system flexibility and, consequently, of system resilience [130]. The species specific to limited utilities exhibit conservation potential. The utilitarian redundancy model (as an analogue to the ecological redundancy hypothesis) determines the use of species richness for the same therapeutic function as a facilitator of the maintenance of indigenous healing systems [131]. Collecting plants with redundant utilities could offset the use pressure to the indigenous populations of restricted habitats and growing only in primary forests.

The total richness and increasing uses of indigenous and non-indigenous species and habitats in indigenous medical systems reveals the dynamism of indigenous systems and an ability to adjust to changes. The acceptance of non-indigenous resources indicates cultural evolution and dynamic indigenous knowledge systems and is considered an adaptive asset. Nonetheless, overall knowledge, cultivation, and maintenance of indigenous medicinal species within rural communities were decreasing as an effect of modernization. Traditional subsistence has undergone change as a result of ever-increasing human population, the gradual change in food habits, and increasing supply and communication [132]. All these changes have transformed and shaped the traditional systems [7] and spurred the uses of secondary forests and non-indigenous species. We believe that usage of non-indigenous species with caution will facilitate conservation of biodiversity and improve livelihoods in the future because they may be more likely than native species to persist and to provide additional services in the areas where climate and land-use changes are evident [130]. Their role in our study area seems profound because climate and land-use changes are already manifested [36]. Harvesting non-indigenous plants from nearby and disturbed areas might help to preserve distant, old-growth and pristine forests. In the pursuits of biodiversity preservation, we agree with the idea of Peretti [133] that if peaceful coexistence in a multicultural society is a valued goal for human, why not for plants, too? Conserving as many plants as possible is important for the benefit of humans and of other species [134] because we believe that every plant has medicinal properties [29].

## Conclusions

Distribution and collection patterns of species with high relative importance and use value showed that accessible areas near vicinity of villages were important for collection. The use of non-indigenous species and nearby habitats as medicinal was gaining ground as primary forests are limited, inaccessible and overexploited, indigenous species declined, and traditional practices were replaced with subsequent knowledge. Change in ecology and land-use, species biology, and sociocultural traditions, along with other factors, have led to the increasing use of secondary forests and non-indigenous species, supporting the ecological apparency hypothesis. We assert that collecting nearby plants with redundant utilities could offset the use pressure on native populations, resulting in conservation of indigenous biota and maintenance of indigenous medicines. The total richness and increasing uses of indigenous and non-indigenous species and habitats reveals the dynamism, fluidity, and resilience of indigenous systems to adjust to future changes. As the non-indigenous species and human-derived landscapes are used cautiously and as compliments to indigenous medical systems, the whole process is considered a part of adaptation, and a valuable asset for ethnobotany.

## Supporting Information

S1 File English and Nepali questionnaires (DOCX)

S1 Table List of 74 plants and their use values (DOCX)
