# Correction: Land-Use and Socioeconomic Change, Medicinal Plant Selection and Biodiversity Resilience in Far Western Nepal

**DOI:** 10.1371/journal.pone.0169447

**Published:** 2016-12-29

**Authors:** The PLOS ONE Staff

## Notice of Republication

This article was republished on December 15, 2016, as a Supporting Information file was published in error. The publisher apologizes for the error. Please download this article again to view the correct version.
