# Supplementary material for: Land-Use and Socioeconomic Change, Medicinal Plant Selection and Biodiversity Resilience in Far Western Nepal
Source: PLoS One. 2016 Dec 9;11(12):e0167812. doi: 10.1371/journal.pone.0167812 (PMC5147989; doi:10.1371/journal.pone.0167812)
Supplement: S1 File — (DOCX) [file pone.0167812.s001.docx]

Survey Questionnaire

**Research topic: Indigenous and nonindigenous plants, Ethnobotany and Biodiversity Conservation**

*I am Ripu Kunwar, a graduate student, pursuing PhD at Florida Atlantic University. I am doing a research work on conservation and sustainable utilization of medicinal plants and indigenous medicines of far western Nepal. Now, I am here in your village to do the research on declining and newly introduced medicinal plants and their roles in indigenous medicine. I have prepared this questionnaire to collect more information about species in decline, newly introduced species in indigenous medicine, why and how were they introduced, and future of this process. Does this species incorporation process compatible to socio-culture, economy and ecology of your area and the future of indigenous medicine as well? I do hereby request you to participate in this survey voluntarily. Feel very much free to express whatever you feel appropriate. We assure you that you are not entitled to receive any benefit or suffer any loss due to what you have expressed in this survey. Your information will be encoded and averaged for interpretation in order to secure the confidentiality. If you feel discomfort while participating in this survey, you may discontinue any time.*

*Thanking you!*

*Researcher*

******************************************************************************************************************

*Name of respondent: Age: Occupation:*

*Address: Sex: Code:*

1. Could you please give us a plant name (s) that you/your relatives used in your time and now has/have been declined/lost from your VDC? Who used that plant most and why?

| SN | Name of Plant | Plant description | When it was seen last time | Where it was found commonly (tick) | | | | |
| --- | --- | --- | --- | --- | --- | --- | --- | --- |
|  |  |  |  | PF | SF | TL | FL | HG |
| 1 |  |  |  |  |  |  |  |  |
| 2 |  |  |  |  |  |  |  |  |
| 3 |  |  |  |  |  |  |  |  |
| 4 |  |  |  |  |  |  |  |  |

PF= Primary wild forest, SF = Secondary, TL = Transition land, FL = Farmland, HG = Home garden

1. Please give us some detail about that/those plants.

| SN | Name of Plant | Who used this plant (ethnic group) | Why did they use it (reasons) | Why was it declined, what were the reasons for declining |
| --- | --- | --- | --- | --- |
| 1 |  |  |  |  |
| 2 |  |  |  |  |
| 3 |  |  |  |  |
| 4 |  |  |  |  |

1. Please give us more detail about that/those plants.

| SN | Name of Plant | Was this plant replaced by another, if yes which one | What did you feel about this replacements (1, 2 ,3, 4, 5) | Are there other plants which have similar characteristics to the declined ones |
| --- | --- | --- | --- | --- |
| 1 |  |  |  |  |
| 2 |  |  |  |  |
| 3 |  |  |  |  |
| 4 |  |  |  |  |

Very much-5, Somewhat-4, Neutral-3, Not much-2, Not at all-1 (Likert scale)

1. Then, could you please give us the name/detail of a newly introduced plant that has now been incorporated in your indigenous medicine?

| SN | Name of Plant | Plant description | When was it seen in the village first time | Where it is found commonly | | | | |
| --- | --- | --- | --- | --- | --- | --- | --- | --- |
|  |  |  |  | PF | SF | TL | FL | HG |
| 1 |  |  |  |  |  |  |  |  |
| 2 |  |  |  |  |  |  |  |  |
| 3 |  |  |  |  |  |  |  |  |
| 4 |  |  |  |  |  |  |  |  |

1. Please give us more detail about that/those plants.

|  |  |  |  |  | Why for | |
| --- | --- | --- | --- | --- | --- | --- |
| SN | Name of Plant | Who is commonly using this (ethnic group) | Why this has been used? | Is this as replacement or just to diversify the stock | Replacement | Diversification |
|  |  |  |  |  |  |  |
| 2 |  |  |  |  |  |  |
| 3 |  |  |  |  |  |  |
| 4 |  |  |  |  |  |  |

1. How compatible this replacement or use is? Very much-5, Somewhat-4, Neutral-3, No tmuch-2, Not at all-1 (Likert scale)

| SN | Name of Plant | Socio-cultural | Economical | Ecological | Future |
| --- | --- | --- | --- | --- | --- |
| 1 |  |  |  |  |  |
| 2 |  |  |  |  |  |
| 3 |  |  |  |  |  |
| 4 |  |  |  |  |  |

1. Please give us five most important medicinal plants and their uses in your life or livelihood?

| SN | Name of Plant | Uses | Indigenous or non | Declining or increasing | Where it was or is found | |
| --- | --- | --- | --- | --- | --- | --- |
|  |  |  |  |  | Past | Present |
| 1 |  |  |  |  |  |  |
| 2 |  |  |  |  |  |  |
| 3 |  |  |  |  |  |  |
| 4 |  |  |  |  |  |  |
| 5 |  |  |  |  |  |  |

1. Additional info if you would you like to add

**a}t8L, 88]Nw'/f / bfr{'nf lhNnfsf hl8a'6L / k/Dk/fut cf}iflwo 1fg tyf k2tLdf b]lvPsf cfofdx?sf] ;dli6ut ljZn]if0f**

gd:sf/ π

lbuf]sf] hl8a'6L ;+/If0f / ltgsf] pkof]u tyf k/Dk/fut cf}iflwo 1fgsf] ;Daw{g d]/f] cWoog cg'zGwfgsf] ?rL xf] . o;} qmddf klZrd g]kfnsf] a}t8L, 88]Nw'/f / bfr{'nf lhNnfsf] k/Dk/fut cf}iflwo 1fg tyf k2tLdf rn]sf, nf]kf]Gd'v tyf nf]k eO{;s]sf hl8a'6LhGo la?jf / cfofltt cyjf ljb]zL lj?jfx?sf] k|efj af/]df cWoog ug{ o; k|ZgfjnL tof/ kf/]sf] x' . of] s'g} Jofj;fosf] lx;fan] ul/Psf] xf]O{g . of] laz'2 z}lIfs p2]Zon] k|]l/t cg'zGwfg xf] / o;af6 k|fKt hfgsf/Lx? clen]v ug]{, k|sfzg ug]{ / ;DalGwt ;+:yfnfO{ l;kmfl/; ug]{ / ;xof]u ug]{ xf] . o;df tkfO{sf] ;xof]u :jo+;]jL x'g]5 . tkfO{nfO{ hl8a'6L ;+/If0f / ltgsf] pkof]u tyf k/Dk/fut 1fg ;DalGw hfgsf/Lx? lbg dg gnfu] jf lard} 6'Ë\ofpg dg nfu] :jo+ lg0f{o ug{ ;Sg'x'g]5 . wGoafb π

cg'zGwfgstf{

**k|ZgfjnL ldtL**

pQ/bftfsf] gfd: jif{: lnË M dlxnf k'?if

uf la ; M 7]ufgf M ;'rs M

1. tkfO{ jf tkfOsf] ufFpsf] s'g} AolStn] ljutdf k|of]u ug]{ u/]sf] la?jf h8La'6L t/ clxn] of] uff=lj=;= jf If]qaf6 x/fP/ uPsf] 5g <_ 5g eg] s'g s'g h8La'6L lj?jf x'g < Tof] h8La'6L lj?jf s;n] lsg k|of]u ub{y] < **3gf h+un, ;fd'bfoLs jg, kfvf] jf v]; uPsf] hldg, v]t jf/L, s/];f jf/L**

|  | Gffd | s:tf] la?jf | slxn];Dd b]lvPsf] lyof] | sxfF k\|z:t kfO{GYof] | | | | |
| --- | --- | --- | --- | --- | --- | --- | --- | --- |
|  |  |  |  | 3h | ;fj | kfvf] | v] jf | s jf |
| ! M |  |  |  |  |  |  |  |  |
| @ M |  |  |  |  |  |  |  |  |
| # M |  |  |  |  |  |  |  |  |
| $ M |  |  |  |  |  |  |  |  |

1. lt la?jf h8La'6Lsf] af/]df yk hfgsf/Lx? _

|  | Gffd | s;n] k\|of]u ub{y] | lsg k\|of]u ub{y] | lsg nf]k ePsf xf]nfg |
| --- | --- | --- | --- | --- |
| ! M |  |  |  |  |
| @ M |  |  |  |  |
| # M |  |  |  |  |
| $ |  |  |  |  |

1. k/Dk/fut cf}iflwo 1fgdf To; la?jf h8La'6Lsf] k|lt:yfkg c? s'g}n] u/]sf] 5 jf ug{ ;S5 _

|  | Gffd | k\|lt:yfkg u/]sf]] la?jfsf] gfd | k\|lt:yfkg tkfO{nfO{ s:tf] nfu]sf] 5 | k\|lt:yfkLt lj?jfsf] h:tf] sfd ug{ ;Sg] cGo lj?jf |
| --- | --- | --- | --- | --- |
| ! M |  |  |  |  |
| @ M |  |  |  |  |
| # M |  |  |  |  |
| $ M |  |  |  |  |

/fdf]| %, l7s} $, ;fdfGo #, g/fdf]| @, x'g'x'b}g ! (Likert scale)

1. To:t} klxnf gb]v]sf] t/ xfn tkfFO{sf] k/Dk/fut cf}iflwo 1fg tyf k2tLdf k|of]u eO{/fv]sf] s'g} la?jf 5 <

|  | Gffd | s:tf] la?jf xf] | Slxn], lsg / s;/L oxfF cfof] | sxfF k\|z:t kfO{G5 | | | | |
| --- | --- | --- | --- | --- | --- | --- | --- | --- |
|  |  |  |  | 3h | ;fj | kfvf] | v] jf | s jf |
| ! M |  |  |  |  |  |  |  |  |
| @ M |  |  |  |  |  |  |  |  |
| # M |  |  |  |  |  |  |  |  |
| $ M |  |  |  |  |  |  |  |  |

1. To; la?jf h8La'6Lsf] af/]sf] yk hfgsf/Lx?

|  | Gffd | s:n] k\|of]u ub}{ cfPsf 5g | s]sf] nfuL k\|of]u ub}{ | k\|of]hg c? s'g}sf] k\|lt:yfkg xf] | Jff cf}iflwo k2tLdf df}hbftsf] nfuL xf] |
| --- | --- | --- | --- | --- | --- |
| ! M |  |  |  |  |  |
| @ M |  |  |  |  |  |
| # M |  |  |  |  |  |
| $ M |  |  |  |  |  |

1. k/Dk/fut cf}iflwo k2tLdf o;sf] k|of]u tkfO{nfO{ s:tf] nfu]sf] 5 < /fdf]| %, l7s} $, ;fdfGo #, g/fdf]| @, x'g'x'b}g ! (Likert scale)

|  | Gffd | k/Dk/fut cf}iflwo k2tLdf o;sf] k\|of]u tkfO{nfO{ s:tf] nfu]sf] 5 | | | | |
| --- | --- | --- | --- | --- | --- | --- |
|  |  | ;fdflhs | ;f:s[lts | Cfly{s | Jfftfj/l0fo | eljio |
| ! M |  |  |  |  |  |  |
| @ M |  |  |  |  |  |  |
| # M |  |  |  |  |  |  |
| $ M |  |  |  |  |  |  |

1. tkfO{n] cfkm\gf] hLjgdf k|of]u ug{'ePsf] s]xL h8La'6Lsf] gfd / ltgsf] d'Vo pkof]u <

|  | Gffd | k\|of]u | Tof] sxfF kfO{G5 jf kfO{GYof] | | /}yfg] jf cfofltt | 36\bf] jf a9\bf] |
| --- | --- | --- | --- | --- | --- | --- |
|  |  |  | klxnf | clxn] |  |  |
| ! | 66]nf] |  |  |  |  |  |
| @ | ;t'jf |  |  |  |  |  |
| # |  |  |  |  |  |  |
| $ |  |  |  |  |  |  |
| % |  |  |  |  |  |  |

1. tkfO{nfO{ hl8a'6L / k/Dk/fut cf}iflwo 1fgsf af/]df eGg dg nfu]sf] yk s'/fx? <

**k|ZgfjnL ldtL**

pQ/bftfsf] gfd: jif{: lnË M dlxnf k'?if

pd]/ ju{ : ufFp e/Ldf ;a} eGbf kfsf], uFfpdf # hgf dWo]df kg]{ kfsf], ufFpdf % hgf dWo]df kfsf]

uf la ; M 7]ufgf M

1. Could you please give us a plant name (s) that you/your relatives used in your time and now has/have been unseen/lost from your VDC? (klxnf tkfO{ jf tkfOsf] ufFpsf] s'g} AolStn] ljutdf k|of]u ug]{ u/]sf] la?jf h8La'6L t/ clxn] of] uff=lj=;= jf If]qaf6 x/fP/ uPsf] 5g <_ 5g eg] s'g s'g h8La'6L lj?jf x'g < lt la?jf h8La'6Lsf] laz]if k|of]u s] s] lyof] <

|  | Gffd | laz]if k\|of]u |
| --- | --- | --- |
| ! M |  |  |
| @ M |  |  |
| # M |  |  |

1. Please give some detail about those plants. (To; la?jf h8La'6Lsf] cGo laz]iftfx? klg atfO lbg'xf]; _

| ! M |  |  |
| --- | --- | --- |
| @ M |  |  |
| # M |  |  |

1. Who used that plant most? (Tof] h8La'6L lj?jf s;n] lsg a9L k|of]u ub{Yof] <

| ! M |  |  |
| --- | --- | --- |
| @ M |  |  |
| # M |  |  |

1. If you used and it is still being used, how did you learn to use that plant? (olb tkfO{n] k|of]u ug'{x'GYof] eg] To;sf] pkof]u ug]{ 1fg sf] af6 s;/L kfpg' eof] <
2. Do you have any idea, how many peer healers are there in your vdc? tkfO{ h:t} h8La'6L k|of]u ul/ k|fylds :jf:Yo pkrf/ ug]{, u/fpg] o; uf la ;df c? slthgf x'g'x'G5 < olb tkfO{n] ug'{x'Gg eg] klg h8La'6L k|of]u ul/ k|fylds :jf:Yo pkrf/ ug]{ o; uf la ; df slthgf x'g'x'G5 <
3. Please let us about the most important medicinal plant and why it is the most important? (clg tkfOsf] lhjgdf jf tkfO{nfO{ nfu]sf] ;a} eGbf dxTjk'0f{ la?jf h8La'6L k|fylds :jf:Yo pkrf/sf] lx;fadf s'g xf] / To;sf] s] k|of]u 5<
4. Then, could you please give me the name of a new plant that you have now seen in your VDC but it was not seen before. (To:t} klxnf gb]v]sf] t/ xfn tkfFO{sf] ufFp tyf j/k/ b]vf k/]sf] s'g} la?jf 5<_

|  | Gffd | slxn] b]lv b]vfk/]sf] |
| --- | --- | --- |
| ! M |  |  |
| @ M |  |  |
| # M |  |  |

1. Is it also useful in ethnomedicine or primary health care? (s] Tof] gofF lj?jfsf] 3/fo;L cf}iflw, h8La'6L k|of]u af/] s]lx yfxf 5<_

|  | Gffd | laz]if k\|of]u |
| --- | --- | --- |
| ! M |  |  |
| @ M |  |  |
| # M |  |  |

1. Who mostly use this and when? -olb 5 eg] s:n] / s] sf]nfuL k|of]u ub}{ cfPsf 5g _

|  | Gffd | s:n] k\|of]u ub}{ | s]sf] nfuL k\|of]u ub}{ |
| --- | --- | --- | --- |
| ! M |  |  |  |
| @ M |  |  |  |
| # M |  |  |  |

1. Why it has been used? -lsg of] k|of]u x'g yfn]sf] 5 <

| ! M |  |  |  |
| --- | --- | --- | --- |
| @ M |  |  |  |
| # M |  |  |  |

1. If its not used in health care, do you have any idea which another new plant (in your life time) is being increasingly used for primary health care in your village? -olb dfly elgPsf lj?jfx? hl8a'6L k|of]usf xf]O{gg eg] s'g gofF la?jf clxn] k|fylds :jf:Yo pkrf/df k|of]u x'g yfn]sf] 5 <
2. More info, eGg dg nfu]sf] yk s'/fx? M
