# Supplementary material for: Land-Use and Socioeconomic Change, Medicinal Plant Selection and Biodiversity Resilience in Far Western Nepal
Source: PLoS One. 2016 Dec 9;11(12):e0167812. doi: 10.1371/journal.pone.0167812 (PMC5147989; doi:10.1371/journal.pone.0167812)
Supplement: S1 Table — (DOCX) [file pone.0167812.s002.docx]

**S 1 Table. Relative Importance and Use Values of Medicinal Plants Used in far western Nepal**

| **SN** | **Voucher code** | **Family name** | **Scientific name** | **Local and common names** | **Habit** | **Indigenous use in study area** | **Plant parts used** | **Use Value** | **Family Use Value** | **Relative Importance** |
| --- | --- | --- | --- | --- | --- | --- | --- | --- | --- | --- |
|  | BBU 057, KU 07273 | Acanthaceae | *Justicia adhatoda* L. | *Vasakha (L), Asuro (N), Basa, Brisha (S)* | Shrub | Leaf juice is used to cure diarrhea and dysentery. | Leaves | 0.037037 | 0.03703 | 0.096531 |
|  | KU 07224 | Adiantaceae | *Adiantum cappillus-veneris* L. | *Gophale, Rasyada (N)* | Herb | Root juice is taken in migraine, snake bite and scorpion sting. Plant is useful in diarrhea, spleen disorders. | Roots | 0.074074 | 0.07407 | 0.289593 |
|  | BKU 098, KU 07223 | Amaranthaceae | *Achyranthes aspera* L. | *Bipya kuro (L), Apamarga, Dattiwan (N), Apamarga, Kharamanjari (S)* | Herb | Root juice is used for cough, common cold, diarrhea and dysentery. | Roots | 0.12963 | 0.12963 | 0.289593 |
|  | KU 07245 | Anacardiaceae | *Rhus parviflora* Roxb. | *Bewoti (L), Satibayer (N), Tintideek (S)* | Shrub | Fruit decoction is taken for doarrhoea and dysentery. | Fruits | 0.037037 | 0.03703 | 0.096531 |
|  | KU 07210 | Apiaceae | *Angelica glauca* L. | *Gannano (N)* | Herb | Dried roots are antihelminthic and useful in gastric, fever, and stomachache. | Roots | 0.574074 | 0.36296 | 0.309201 |
|  | Eristoff 605 |  | *Carum carvi* L. | *Kaljira (N)* | Herb | Plant is useful in cold, cough and fever. | Fruits | 0.814815 |  | 0.193062 |
|  | Schmidt 2949 |  | *Carum species* | *Jangali jira (L)* | Herb | Fruits are applied against swelling of breast and testicles. | Fruits | 0.074074 |  | 0.096531 |
|  | DBU 006, KU 07276 |  | *Centella asiatica* (L.) Urb. | *Khochade (L), Ghodtapre, (N), Brahmi, Madukparni (S)* | Herb | Leaf juice is used in urinary problems and cuts and wounds and for memory longevity. | Leaves | 0.111111 |  | 0.289593 |
|  |  |  | *Eryngium foetidum* L. | *Ban dhaniya (L)* | Herb | Stomachache, indigestion, Asthma | Leaves | 0.240741 |  | 0.21267 |
|  | BKU 176, KU 07234 | Araceae | *Acorus calamus* L. | *Sutak, Charila (L), Bojho (N), Wacha, Ugragandha (S)* | Herb | Rhizome juice is anthelminthic and pesticidal in properties. The juice is given to stomachache and trunk pain. It is also considered to increase memory longevity. | Rhizome | 0.037037 | 0.05555 | 0.405732 |
|  | KU 562/00 |  | *Arisaema flavum* (Forsk.) Schott | *Banko (L, N)* | Herb | Rhizome juice is applied on earache and skin diseases. Young shoots are cooked as vegetable. | Rhizome | 0.074074 |  | 0.096531 |
|  | KU 07264, BBU 054 | Asclepiadaceae | *Calotropis gigantea* (L.) Dryand. | *Aak (L, N), Ark, Alarka (S)* | Shrub | Latex is useful in arthritis, inflammation and keeping out thorns from wounds. It is also applied on wasp sting. | Stem latex | 0.037037 | 0.03703 | 0.386124 |
|  | Henry 12260 | Asteraceae | *Acmella calva* (DC.) Jansen | *Marethi (L, N)* | Herb | Plant paste is used against snake bite. Inflorescence is used as appetizer. | Flowers and fruits | 0.037037 | 0.22685 | 0.193062 |
|  | Abott 12931 |  | *Ageratina adenophora* (Spreng.) King & H.Rob. | *Banmara (L, N)* | Herb | Leaf extract is used for bleeding control. | Leaves | 1.425926 |  | 0.096531 |
|  | Heng 8512 |  | *Ageratum conyzoides* L. | *Gandhe, Nilgandhe Kalo jhar (L, N), Visamusti, Osari (S)* | Herb | Stem juice is useful in bleeding control. | Stem | 0.055556 |  | 0.096531 |
|  | KU 07214 |  | *Artemisia vulgaris* L. | *Kurje pati (L), Titepati (N), Surparnaa, Nakuli, Nagadamni, Damanaka (S)* | Herb | Plant is used in headache, fever and it is also used as insecticide. Leaves are used in skin itching and scabies. | Leaves | 0.074074 |  | 0.289593 |
|  | KU 07222 |  | *Cirsium verutum* (D.Don) Spreng. | *Thakil, Dhande kanda (L), Thakailo (N)* | Herb | Root is used as refresher and for calmness. It is also applied for stomachache and abdominal pain. | Roots | 0.074074 |  | 0.21267 |
|  | BKU 055, KU 07226 |  | *Eclipta prostrata* L. | *Bhringraj (N), Kesaranjan, Bhringraj (S)* | Herb | Leaf juice is given for dyspepsia and is applied for scorpion sting. Root is used as tonic for liver and hair growth. | Leaves | 0.037037 |  | 0.309201 |
|  | KU 07208 |  | *Elephantopus scaber* L. | *Gomukhi (L, N), Satamulika (S)* | Herb | Root is given to control vomiting. | Roots | 0.037037 |  | 0.096531 |
|  | D’Arcy 341a |  | *Xanthium strumarium* L. | *Musekuro, Sangekuro (L), Bhede kuro (N), Sankesvara, Arista (S)* | Herb | Seed powder is useful in earache, dysentery and skin diseases. | Seed | 0.074074 |  | 0.289593 |
|  | BKU 061, KU 07238 | Bignoniaceae | *Oroxylum indicum* (L.) Kurz | *Sanna, Tatelo (N), Shyonaka (S)* | Tree | Root decoction is used in diarrhoea and dysentery. Seeds are digestive for livestock. | Roots | 0.037037 | 0.03703 | 0.193062 |
|  | BBU 105 | Bombacaceae | *Bombax ceiba* L. | *Simal (N), Moca, Salmali (S)* | Tree | Flowers and seeds are used in dysentery. Root, bark and seeds are emetic and stimulant. | Flowers, Seeds | 0.037037 | 0.03703 | 0.193062 |
|  | Hoffmeister 78a | Caryophyllaceae | *Drymaria cordata* (L.) Willd. ex Roem. & Schult. | *Abijalo (N)* | Herb | Leaf is used as calmness, fresh and for cough. | Leaves | 0.074074 | 0.74074 | 0.193062 |
|  | KU 07280 | Combretaceae | *Terminalia bellerica* (Gaertn.) Roxb | *Barro (N), Aksha (S)* | *Tree* | Fruits are useful in stomach disorders and cough. If taken excessive, cause vomiting. Fruit decoction is given to livestock for diarrhea. | Fruits | 0.037037 | 0.037037 | 0.289593 |
|  | BKU 068, KU 07278 |  | *Terminalia chebula* Retz. | *Sele, Harado (L), Harra (N), Avaya, Haritaki (S* | Tree | Infusion of fruits is useful in cough and cold. | Fruits | 0.037037 |  | 0.096531 |
|  | KU 07265 | Convolvulaceae | *Cuscuta reflexa* Roxb. | *Aakasbeli (N), Amaruela (S)* | Climber | Plant paste is effective for headache, bodyache and itches. Stem decoction for jaundice, fever | Stem | 0.444444 | 0.44444 | 0.309201 |
|  |  | Cucurbitaceae | *Trichosanthes tricuspidata* Lour. | *Indreni (L, N)* | *Climber* | Fruit juice is useful in otitis and rhinitis, asthma, rheumatism, stomachache. | Fruits | 0.203704 | 0.20370 | 0.309201 |
|  | Heng 8615 | Davalliaceae | *Nephrolepis cordifolia* (L.) K. Presl | *Pani amala (N), Rasmada (L)* | Herb | Refresher, galactogogue | Rhizome | 0.12963 | 0.12963 | 0.193062 |
|  |  | Euphorbiaceae | *Euphorbia royleana* Bioss. | *Siudi (L, N), Snuhi (S)* | Shrub | Stem latex is used in joint pain/leg pain and eye complaints. | Stem latex | 0.37037 | 0.24074 | 0.193062 |
|  | BBU 056, KU 07260 |  | *Jatropha curcas* L. | *Inna (L), Sajiwan (N), Vyaghra eranda (S)* | Shrub | Seed oil is applied for arthritis and boils. Bark juice is useful in wounds, scabies and ringworm. | Seed oil | 0.111111 |  | 0.21267 |
|  | KU 07209 | Fabaceae | *Abrus precatorius* L. | *Ratigedi (N), Gunja (S)* | Shrub | Fruits are used in catarrhal diseases and as arbortfacient. | Fruits | 0.074074 | 0.06481 | 0.193062 |
|  | Miller 7018 |  | *Bauhinia variegata* L. | *Koiralo (N), Kachnar, Kovidarah (S)* | Tree | Flower and floral buds are eaten regularly to cure leucorrhoea and mumps. | Flowers | 0.037037 |  | 0.096531 |
|  | BKU 059, KU 07227 |  | *Mucuna pruriens* (L.) DC. | *Kauchho (N), Kapikachhu (S)* | Climber | Roots are tonic and stimulant. | Roots | 0.037037 |  | 0.193062 |
|  |  |  | *Saraca asoka* (Roxb.) Wilde | *Asoka (L)* | Tree | Menstruation, bark for stomachache | Bark | 0.111111 |  | 0.193062 |
|  |  | Fagaceae | *Quercus lanata* Sm. | *Latyaz (L), Baanjh (N)* | Tree | Heart wood is taken as tea and it is laxative in nature. | Woods | 0.037037 | 0.03703 | 0.096531 |
|  | DBU 067, KU 07277 | Gentianaceae | *Swertia chirayita* (Roxb. ex Fleming) | *Lek tite (N), Kirat, Bhunimwa (S)* | Herb | Whole plant is used for cough and fever. | Whole plant | 0.037037 | 0.03703 | 0.096531 |
|  | KU 563/00 | Hippocastanaceae | *Aesculus indica* (Colebr. ex Cambess.) Hook. | *Panger, Karu (N), Naaru (S)* | Tree | Seed oil is valued for joint pain and skin problems. | Seed oil | 0.037037 | 0.03703 | 0.193062 |
|  | BKU 020, KU 07253 | Hypoxidaceae | *Curciligo orchoides* Gaertn. | *Kalmusali (N), Talmule (S)* | Herb | Root is used as tonic and effective on paralysis. | Roots | 0.037037 | 0.03703 | 0.193062 |
|  | DBU 099, KU 07272 | Juglandaceae | *Juglans regia* L. | *Okhar (N), Akshotaka (S)* | Tree | Bark is used in scabies, allergy and toothache. | Bark | 0.037037 | 0.03703 | 0.21267 |
|  | BKU 058 | Lamiaceae | *Mentha spicata* L. | *Pudina (L), Tulsi pate (N), Putitha (S)* | Herb | Plant is used for freshness, asthma and urinary complaints. | Leaves | 0.037037 | 0.12962 | 0.289593 |
|  | KU 07200 |  | *Ocimum gratissimum* L. | *Bantulasi (L), Babari (N)* | Herb | Fresh leaves are used as tea. Whole plant is taken for diuretic, asthma and rheumatism. | Leaves | 0.222222 |  | 0.21267 |
|  | KU 07215 | Lauraceae | *Cinnamomum tamala* (Buch.-Ham.) Nees & Eberm. | *Tejpat (N), Bahugandh, Tachula (S)* | Tree | Leaf is used in gastric problems. Bark is useful in checking nausea and vomiting. | Leaves | 0.074074 | 0.07407 | 0.193062 |
|  | Gentry 22717 | Liliaceae | *Aloe vera* (L.) Burm .f. | *Hattibar (L), Ghiukumari (N), Ghritkumari (S)* | Shrub | Plant leaf is used to get relief from burning. | Leaves | 0.648148 | 0.48148 | 0.096531 |
|  | KU 07256 |  | *Paris polyphylla* Smith | *Satuwa (N), Haimavati, Satuwa (S)* | Herb | Root is used in epilepsy, shock, fever, vomiting control. Its paste is applied on snake bite. | Roots | 0.314815 |  | 0.289593 |
|  | KU 07285 | Lythraceae | *Woodfordia fruticosa* (L.) Kurz | *Dhula, Dhuniya (L), Dhainyaro (N), Agnijwala, Tamra pushpi, Dhataki (S)* | Shrub | Flowers are antipyretic and used in dysentery. | Flowers | 0.074074 | 0.07407 | 0.193062 |
|  |  | Meliaceae | *Melia azedarach* L. | *Neem (N), Aristha, Nimbah (S)* | Tree | Both raw and dried leaves are used for fever and blood disorders. | Leaves | 0.925926 | 0.92592 | 0.193062 |
|  | BKU 023 | Menispermaceae | *Cissampelos pareira* L. | *Batulpate, Gurjegano, Tito belo (N), Ambashtha (S)* | Climber | Root is anthelminthic and antipyretic. Root juice cures headache, stomachache, asthma and urinary problems of livestock. | Roots | 0.037037 | 0.05555 | 0.405732 |
|  | DKU 088 |  | *Tinospora sinensis* (Lour.) Merr. | *Gurjo (N), Guduchi, Amritavali (S)* | *Climber* | Dilute stem juice is drunk for diabetes. | Stem | 0.074074 |  | 0.193062 |
|  | Townesmith 1784 | Myrtaceae | *Psidium guajava* L. | *Ambak (L), Amba, Belauti (N), Amratphala, Peruk, Mamsala (S)* | Tree | Fruit is laxative, colic, astringent to bowls and beneficial to constipation. | Fruit | 0.037037 | 0.03703 | 0.309201 |
|  | BBU 085, KU 07283 |  | *Syzygium cumini* (L.) Skeels | *Jamun (N), Brahaspati, Jambhu (S)* | Tree | Seed powder and bark decoction is used in diarrhea, dysentery, diabetes and inflammatory activity. | Seeds | 0.037037 |  | 0.21267 |
|  |  | Not identified | Not identified | *Shilajeet (N)* | Others | Cuts and wounds, Fever diuretic | Others | 0.462963 | 0.15123 | 0.289593 |
|  |  |  | Not identified | *Bhangkhaper (L)* | Herb | Stomachache, cold and cough | Roots | 0.1296296 |  | 0.193062 |
|  |  |  | Not identified | *Ghiwa jara (L)* | Herb | Gastric | Roots | 0.1296296 |  | 0.096531 |
|  |  |  | Not identified | *Bakar belo (L)* | Herb | Chito pareko | Roots | 0.0740741 |  | 0.096531 |
|  |  |  | Not identified | *Kujar (L)* | Herb | Diphtheria | Roots | 0.0740741 |  | 0.096529 |
|  |  |  | Not identified | *Bal Pakh (L)* | Herb | Tonic | Roots | 0.037037 |  | 0.096531 |
|  | KU 07294 | Orchidaceae | *Pleione humulis* (Sm.) D.Don, | *Manidana (L), Bhuisungava (N)* | Herb | Plant pseudobulb is used as galactogogue for livestock. | Rhizome | 0.037037 | 0.03703 | 0.096531 |
|  | Anderson 399 | Oxalidaceae | *Oxalis corniculata* L. | *Chalmaro (L), Chari amilo (N), Changeri, Amla patrika (S)* | Herb | Leaves are stomachic and useful for throat pain. | Leaves | 0.074074 | 0.07407 | 0.193062 |
|  |  | Poaceae | *Desmostachys bipinata* | *Kush (L), Khas (N), Usirah, Sugandhimulah (S)* | *Herb* | Root juice is useful in purifying blood and cough and cold. | Roots | 0.074074 | 0.07407 | 0.193062 |
|  |  | Polygonaceae | Rumex species | *Sim jada (L)* | Herb | Fever, Cold and cough, Stomachache | Roots | 0.092593 | 0.09259 | 0.289593 |
|  | KU 508/00 | Ranunculaceae | *Delphinium brunonianum* Royle | *Maure (L, N)* | Herb | Dried roots are valued in toothache and cut and wounds. | Roots | 0.037037 | 0.03703 | 0.21267 |
|  | BKU 082 | Rhamnaceae | *Zizyphus mauritiana* Lam. | *Bewari (L), Bayer (N), Badar (S)* | Shrub | Root paste is applied for diarrhea, dysentery and vomiting. Fruits are useful in fever and digestion. | Roots, Fruits | 0.037037 | 0.03703 | 0.289593 |
|  | KU 07242 | Rosaceae | *Fragaria nubicola* Lindl. | *Bhuikafal (N)* | Herb | Fruit paste heals skin diseases and wounds. | Fruits | 0.037037 | 0.03703 | 0.116139 |
|  | KU 07225 | Sapotaceae | *Aesandra butyracea* (Roxb.) Baehni | *Chiura (L), Chiuri, Chiura (N)* | Tree | Oil cake is used to escape out snake, and it can be used as fish poisoning. Oil or ghee is taken to cure cracked heels and lips. Root juice is useful in dysentery. | Seed oil, roots | 0.074074 | 0.07407 | 0.289593 |
|  | KU 07231 | Saxifragaceae | *Astilbe rivularis* Buch.-Ham. ex D.Don | *Sutkeribelo (L), Thulo okhati, Budhookhato (N)* | Herb | Root juice is used for easy delivery during child birth. It is valued for diarrhea, dysentery and hemorrhage. | Roots | 0.037037 | 0.10185 | 0.21267 |
|  | DKU 124, KU 07252 |  | *Bergenia ciliata* (Haw.) Sternb.f. | *Silphode, Vedaite (L), Pakhanved, Dhungephool (N), Asmahan, Asyavedak, Pashanveda (S)* | Herb | Rhizome and root is used in diarrhea, dysentery, gallstone and gastritis. Useful in bleeding control. | Roots | 0.166667 |  | 0.21267 |
|  | DKU 094 | Solanaceae | *Datura stramonium* L. | *Dhatura (N), Kanak, Dhatura (S)* | Herb | Fruits are used as sedative. Seed paste is employed in boils. | Fruits | 0.074074 | 0.07407 | 0.193062 |
|  | KU 72207 |  | *Solanum nigrum* L. | *Nipeni, Ninauni* (L) | Herb | Dysentery | root | 0.074074 |  | 0.096531 |
|  | DKU 086 |  | *Solanum surattense* Burm.f. | *Jhyaure bhanta, Jware baigan (L), Kantakari (N), Kantakari (S)* | Herb | Seed infusion is taken for toothache. | Seeds | 0.074074 |  | 0.096531 |
|  | Heng 8548 | Urticaceae | *Debregeasia longifolia* (Burm.f.) Wedd. | *Githi, Tashiari (L), Daar (N)* | Tree | Wood is used to prepare wooden pots for storing milk and curd which is supposed to be useful for indigestion and sprains. Tender shoots are used as vegetable. | Wood | 0.074074 | 0.07407 | 0.193062 |
|  | KU 07290 |  | *Urtica dioica* L. | *Sisnu (N), Agni damani (S)* | Herb | Stem and wood are used for fracture and sprains. Leaf is used as vegetable. Root juice is given for gastric problems and maintaining blood pressure. | Wood, Leaves, Roots | 0.074074 |  | 0.21267 |
|  | DKU 084, KU 07279 | Valerianaceae | *Valeriana jatamansii* Jones | *Juge jadi, Simme, Samayo (L), Sugandhwal (N), Tagarah, Nataha, Washim (S)* | Herb | Root is anthelmintic and tonic in properties. | Roots | 0.037037 | 0.03703 | 0.193062 |
|  | Weigend 5370 | Verbenaceae | *Lantana camara* L. | *Kande (L), Banmasa (N)* | Shrub | Leaves extract for bleeding control and useful in fever | Leaves | 0.055556 | 0.05555 | 0.193062 |
|  | KU 07259 | Zingiberaceae | *Curcuma angustifolia* Roxb. | *Sathi, Kachur (L), Haldi, Kalo besar (N) Ban haldi, Haridra, Harita (S)* | Herb | Rhizome paste is externally applied for paralysis and useful in asthma and chaskeko. | Rhizomes | 0.092593 | 0.09259 | 0.21267 |

L = Local, N = Nepali, S = Sanskrit
